# Equipment-Free Fabrication of Thiolated Reduced Graphene Oxide Langmuir–Blodgett Films: A Novel Approach for Versatile Surface Engineering

**DOI:** 10.3390/molecules29112464

**Published:** 2024-05-23

**Authors:** Injoo Hwang, Ki-Wan Jeon

**Affiliations:** 1 Department of Mechanical Engineering, Silla University, Busan 46958, Republic of Korea; hwanginjoo@silla.ac.kr; 2 Department of Advanced Technology and Engineering, Graduate School, Silla University, Busan 46958, Republic of Korea; 3 Department of Fire Protection & Safety Management, Silla University, Busan 46958, Republic of Korea

**Keywords:** thiolated reduced graphene oxide, Langmuir–Blodgett films, functionalized thin film

## Abstract

This research presents a novel method for the fabrication of mercapto reduced graphene oxide (m-RGO) Langmuir–Blodgett (LB) films without the need for specialized equipment. The conventional LB technique offers precise control over the deposition of thin films onto solid substrates, but its reliance on sophisticated instrumentation limits its accessibility. In this study, we demonstrate a simplified approach that circumvents the necessity for such equipment, thereby democratizing the production of m-RGO LB films. Thiolation of reduced graphene oxide (rGO) imparts enhanced stability and functionality to the resulting films, rendering them suitable for a wide range of applications in surface engineering, sensing, and catalysis. The fabricated m-RGO LB films exhibit favorable morphological, structural, and surface properties, as characterized by various analytical techniques including scanning electron microscopy (SEM), X-ray diffraction (XRD), and Fourier-transform infrared spectroscopy (FTIR). Furthermore, the performance of the m-RGO LB films is evaluated in terms of their surface wettability, electrochemical behavior, and chemical reactivity. The equipment-free fabrication approach presented herein offers a cost-effective and scalable route for the production of functionalized graphene-based thin films, thus broadening the scope for their utilization in diverse technological applications.

## 1. Introduction

Electrically conductive glass electrodes, renowned for their transparency, play a vital role in numerous photoelectronic applications [1]. Indium tin oxide (ITO) and fluorine tin oxide (FTO) have emerged as the preferred materials for such electrodes in contemporary research and industrial practices. However, their utilization faces significant challenges, primarily stemming from the limited availability of indium, susceptibility to degradation in acidic and alkaline environments, reduced transparency in the near-infrared (IR) range, and vulnerability to ion diffusion within polymer layers [2]. Graphene has emerged as a promising alternative for electrode materials, thanks to its outstanding optical transmittance of 97.7% for a single layer at 550 nm, combined with high electrical conductivity [3,4]. While graphene films can be produced through methods such as chemical vapor deposition (CVD) and epitaxial growth on silicon carbide (SiC), these techniques are constrained by the requirement for specialized substrates and limitations in scalability and cost-effectiveness [5,6].

An alternative approach to graphene film production involves utilizing either graphene suspension or reduced graphene oxide (RGO) suspension [7,8]. However, the low yield of graphene suspension obtained through ultrasonication renders this method impractical for large-area film fabrication. Conversely, stable colloidal suspensions of graphene oxide (GO) in water can be readily achieved via ultrasonication, facilitated by the presence of hydrophilic oxygen functional groups. Various techniques for preparing GO thin films, including drop-casting, spin-coating, dip-coating, spraying, vacuum filtration, and Langmuir–Blodgett (LB) assembly, have been developed [9,10,11,12,13,14]. Nevertheless, these GO films require extensive chemical or thermal reduction to confer electrical conductivity. Alternatively, RGO films can be directly fabricated from RGO suspension through vacuum filtration [15].

The self-assembly process at liquid–air and liquid–liquid interfaces offers another route for the formation of GO, RGO, and graphene thin films. For example, at the pentane–water interface, graphene oxide film can be generated through the evaporation of pentane by rapidly introducing ethanol into a GO suspension [16]. Similarly, graphite oxide membranes are obtained through the evaporation of GO dispersed in water. However, as-fabricated GO films or membranes require chemical or thermal reduction for electrical conductivity, often involving tedious solvent evaporation steps. Although graphene thin film formation can be directly achieved at the chloroform–water interface following sonication of the mixture, the minimal dispersion of graphene in chloroform makes it unsuitable for large-area film production [17]. Additionally, the susceptibility of thin films to cracking during transfer onto desired substrates presents challenges for achieving continuous large-area thin films. Conversely, RGO thin film fabrication at the water–air interface involves the chemical reduction of a GO suspension [18]. During this process, the majority of RGO sheets precipitate to the bottom, with only a minimal quantity floating on the water to form the RGO thin film. However, this method also proves inadequate for large-area RGO film production on water.

The development of alternative fabrication methods is essential for generating continuous large-area conducting thin films, facilitating the widespread utilization of graphene thin films across diverse application fields. The proposed fabrication approach should address key concerns, including minimizing active material loss and eliminating the need for post-treatments such as chemical or thermal reduction. Recently, our research group reported the equipment-free fabrication of m-RGO LB films. Alongside our research efforts, this study systematically explores the solvent’s influence on the formation of homogeneous m-RGO Langmuir–Blodgett (LB) films. This method capitalizes on a homogeneous dispersion of m-RGO in ethanol. Importantly, the synthesis of m-RGO was previously detailed in our prior work. This fabrication process offers several advantages: flexibility regarding size, shape, and substrate material, avoidance of additional reduction steps, minimal active material loss, and no requirement for specialized instrumentation during fabrication.

## 2. Results and Discussion

### 2.1. The Impact of Organic Solvents on m-RGO Langmuir Film Formation

**Figure 1:**
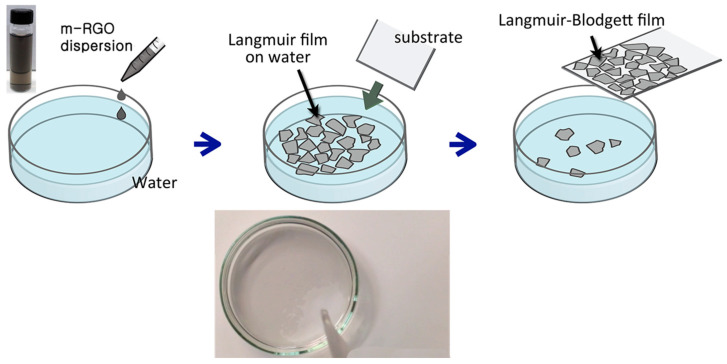
Schematic illustration of the procedure for fabricating mercapto reduced oxide (m-RGO) Langmuir–Blodgett (LB) films.

The schematic m-RGO LB film fabrication procedure is depicted in Figure 1. The fabrication of m-RGO LB film can be achieved through two distinct methods: the dropping and scooping method, or the water draining method. However, for clarity and brevity, we exclusively present the schematic illustration of the scooping method in Figure 1. Initially, upon the sequential addition of m-RGO dispersion onto the water surface, individual m-RGO sheets were formed, exhibiting free movement due to their thin and small dimensions, rendering them difficult to discern. Subsequently, after approximately 0.3 mL of m-RGO dispersion was deposited, the m-RGO sheets became distinctly visible, forming a thin film that covered a portion of the water surface. The size of the m-RGO film exhibited gradual augmentation with the incremental addition of m-RGO dispersion. Upon reaching a discernible threshold visible to the naked eye, the thickness of the resultant m-RGO Langmuir film stabilized at approximately 5~6 nm. Upon full coverage of the water surface by the m-RGO Langmuir film, facile transferability to diverse substrates, including quartz glass, conducting substrate, and polymer, was readily achieved. We have also explored the solvent effect on m-RGO LB film fabrication, and the findings are presented in Table 1. To comprehensively investigate the influence of solvent on m-RGO Langmuir film formation at the water–air interface, we conducted experiments using various organic solvents. Our findings reveal that the formation of m-RGO Langmuir films is attributed to the Marangoni convection. Furthermore, achieving high-quality m-RGO Langmuir films requires a significant difference in surface tension compared to water, along with a lower density than water.

### 2.2. Elemental and Functional Group Analysis of m-RGO

**Figure 2:**
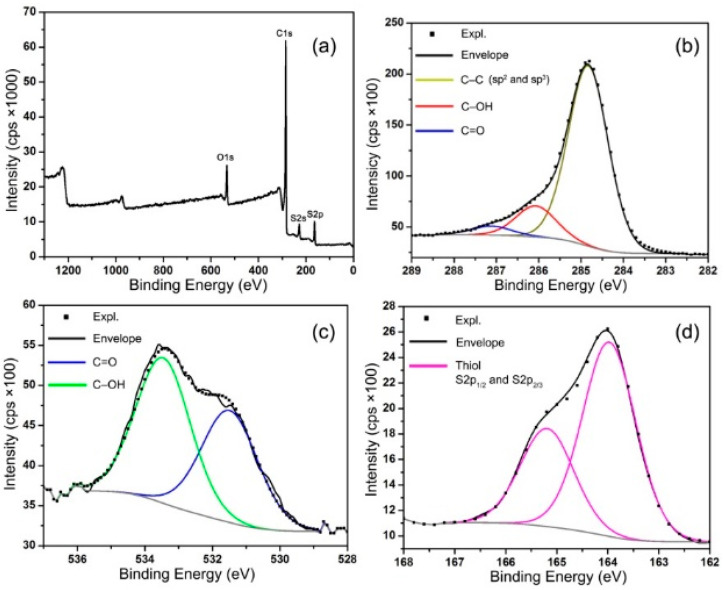
X-ray photoelectron spectroscopy (XPS) spectra of m-RGO: (**a**) wide scan, (**b**) high-resolution C1s, (**c**) high-resolution O1s, and (**d**) high-resolution S2p.

The XPS survey scan and high-resolution C1s, O1s, and S2p spectra of m-RGO are presented in Figure 2. Following the synthesis of m-RGO, elemental analysis was conducted using X-ray photoelectron spectroscopy (XPS). The estimated C:O:S atomic ratio of m-RGO from the XPS wide scan, shown in Figure 2a, is 13:1:0.9, indicating the complete removal of all byproducts and unreacted P_4_S_10_. Additionally, the high-resolution C1s XPS spectrum suggests significant restoration of sp^2^-carbon species in m-RGO. The C–S binding energy (285.3 eV) is very close to that of sp3-hybridized carbon species, with the binding energy difference being less than the XPS resolution limit (0.4 eV), resulting in the C–S bond not being resolved [19]. There is no doubt that m-RGO was reduced during synthesis, as evidenced by both the atomic ratios of m-RGO and the high-resolution C1s XPS spectrum. Both the high-resolution C1s and O1s XPS spectra demonstrate that the obtained m-RGO still harbors oxygen functional groups, including hydroxyl and carbonyl groups. Confirmation of the presence of only thiol functional groups in the as-synthesized m-RGO is provided by the high-resolution S2p XPS spectrum depicted in Figure 2d. The high-resolution S2p XPS spectrum exhibits two distinct peaks attributable to the spin-orbit coupling effect.

**Figure 3:**
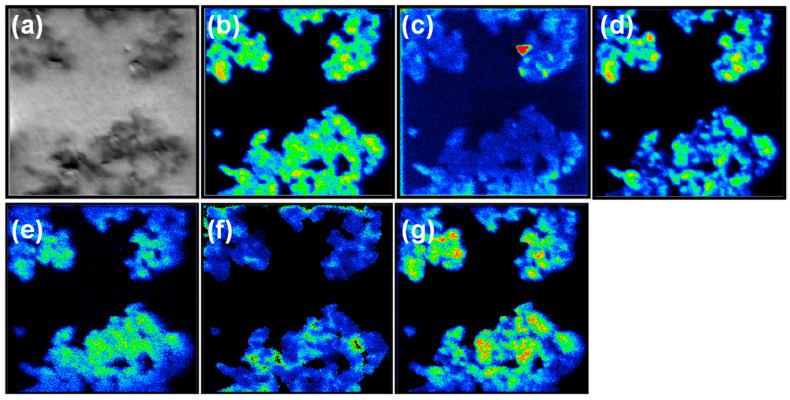
Secondary electron image (**a**) and ion images of (**b**) 12C-, (**c**) 16O-, and (**d**) 32S-, and atomic ratio images of (**e**) C/O, (**f**) C/S, and (**g**) S/O. Color changes from blue through green to red indicate increasing intensity. The nano-secondary ion mass spectrometry (NanoSIMS) and elemental ratios mapping image was obtained using a 25 × 25 μm^2^ area on a m-RGO LB film on an Au substrate.

To gain a deeper understanding of the m-RGO LB film, NanoSIMS analysis was conducted to elucidate the distribution of each element and their relative elemental ratios within the film. The NanoSIMS was employed to examine the distribution of carbon, oxygen, and sulfur within the m-RGO LB film. NanoSIMS, a new-generation double-focusing mass spectrometer characterized by high spatial resolution (up to 50 nm using the Cs+ primary beam and approximately 200 nm with the O-beam), was instrumental in investigating the films fabricated in this study. In Figure 3, the last 8–12 layers were added to generate summed ion images. These images clearly depict the distribution of oxygen and sulfur functional groups on the m-RGO LB film. Although individual elements could not be resolved, spatially correlated hotspots rich in oxygen and sulfur (denoted as (f) and (g) in Figure 3) directly illustrate high-density sulfur functional groups tightly bound to carbon. The elemental ratios mapping image reveals sulfur-rich areas concerning oxygen, strongly indicating the preservation of sulfur functional groups after sonication and assembly of the m-RGO LB film.

### 2.3. Optical and Electrical Characterization of m-RGO LB Film

**Figure 4:**
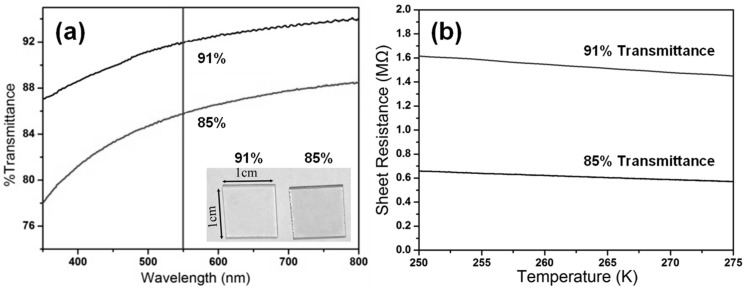
Transmittance (**a**) and sheet resistance (**b**) of m-RGO LB films on slide glass, with corresponding photos of the m-RGO LB film (inset in (**a**)).

The investigation of transmittance and sheet resistance of m-RGO LB films was conducted to elucidate their optical and electrical properties, as well as to establish the correlation between transparency and sheet resistance. The transmittance curve of the m-RGO LB film and the corresponding digital camera photo are depicted in Figure 4a. The m-RGO LB films exhibit a uniform optical transmittance profile across the visible light and near-infrared regions. Thicker m-RGO LB films absorb more light, resulting in lower transparency compared to thinner m-RGO LB films. Transparency measurements of m-RGO LB films were conducted at a wavelength of 550 nm incident light, revealing transmittance values of 91% and 85%, respectively. Considering that each layer of graphene can reduce transmittance by approximately 2.3%, the average number of layers in m-RGO sheets achieving 91% and 85% transmittance is estimated to be around 4 and 7 layers, respectively [20]. The sheet resistance of the m-RGO LB film deposited on slide glass was measured using the Van der Pauw four-probe method. For films exhibiting transmittance levels of 85% and 91%, the sheet resistance is approximately 0.6 MΩ/sq and 1.5 MΩ/sq, respectively, at room temperature. The sheet resistance of the film is significantly influenced by the physical contact of each graphene sheet with the substrate, leading to a notable reduction in sheet resistance for slightly thicker m-RGO films. Moreover, additional heat treatment can substantially enhance the electrical conductivity of reduced graphene oxide films. Consequently, there is potential for further reduction in the sheet resistance of m-RGO LB films.

### 2.4. Structural Analysis of m-RGO Langmuir Film on Water and m-RGO LB Film

**Figure 5:**
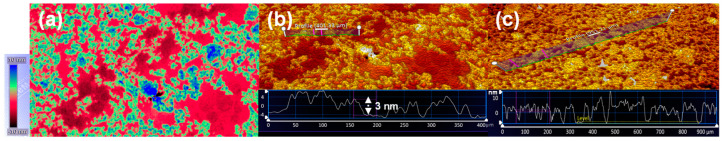
Optical profilometry images of (**a**) and (**b**) m-RGO Langmuir film on water and (**c**) m-RGO LB film on glass. The red color in (**a**) represents water.

To comprehensively analyze the overall morphology, thickness, and root mean square (RMS) roughness of both m-RGO Langmuir film on water and m-RGO LB film on glass, optical profilometry was utilized. The profilometry image of both m-RGO Langmuir film and m-RGO LB film is presented in Figure 5. Both films exhibit an RMS roughness of approximately 1 nm, suggesting a remarkably smooth surface for both the Langmuir film on water and the LB film on glass. It is observed that thicker areas are present on both m-RGO films, likely due to the aggregation of m-RGO sheets. The general thickness of both films, whether on water or glass, is approximately 3 nm. Examination of the profilometry images indicates homogeneous distribution of both m-RGO Langmuir and LB films on the surfaces of water and glass at a macroscopic level. Based on the optical profilometry images, the morphology of the m-RGO Langmuir film remained intact during the transferring process. This underscores the importance of achieving high-quality m-RGO Langmuir film, as it directly contributes to the production of homogeneous and transparent m-RGO LB film over large areas.

**Figure 6:**
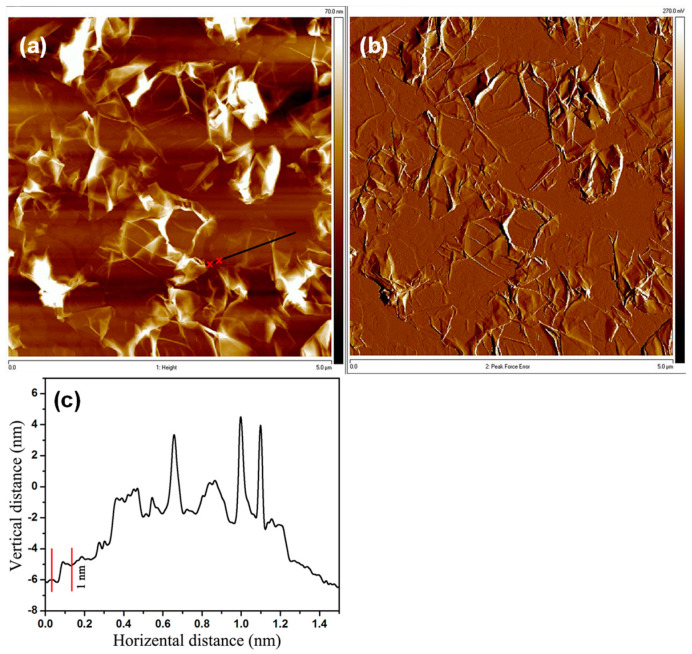
AFM height (**a**) and amplitude (**b**) images of m-RGO LB film on mica, with the corresponding height profile (**c**).

For a microscopic examination of the morphology of the m-RGO LB film, atomic force microscopy (AFM) was employed to investigate the arrangement of each m-RGO sheet on the substrate. Nearly all m-RGO sheets in Figure 6a were observed to be thoroughly exfoliated and evenly distributed on the mica substrate. Additionally, each m-RGO sheet exhibited characteristic wrinkling and folding, consistent with the typical behavior of reduced graphene oxide LB films. Some instances of stacked m-RGO sheets were also observed. The thickness of the m-RGO sheets was measured to be approximately 1 nm in Figure 6c, indicating complete exfoliation. Height profiles revealed occasional spikes and thicknesses exceeding 1 nm, attributed to the presence of wrinkles, folds, and/or stacked m-RGO sheets. Additionally, understanding the degree of deficiency in m-RGO is crucial as it reflects the extent of functionalization of RGO. Raman spectroscopy provides direct insight into the degree of deficiency of graphene by comparing the intensity of the D and G bands. Typically, graphene oxide exhibits a high I_D_/I_G_ ratio (typically higher than 1) due to the presence of various oxygen functional groups on both the basal plane and at the edges. However, the I_D_/I_G_ ratio of RGO decreases (typically lower than 1) due to the restoration of sp2-hybridized carbon species during the reduction process.

**Figure 7:**
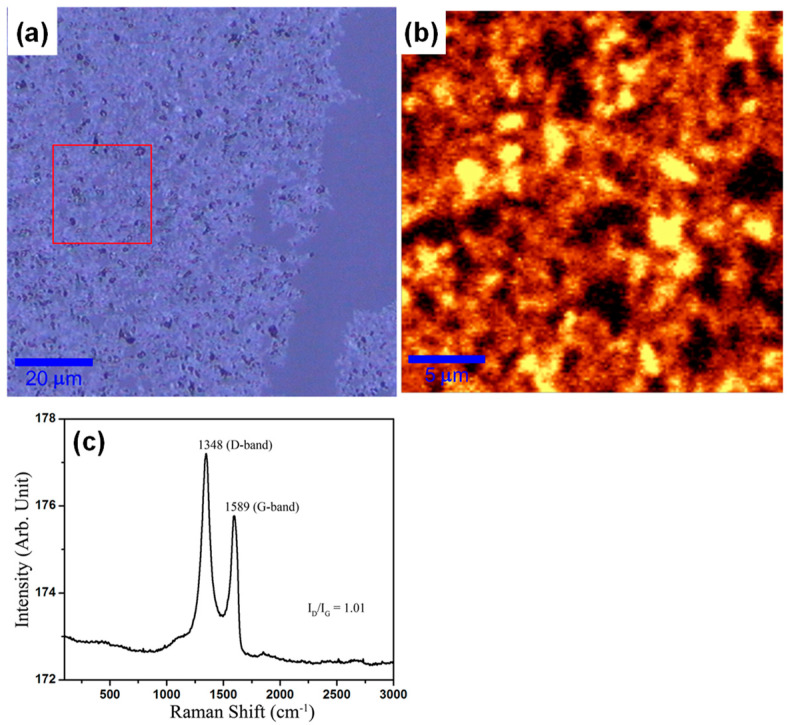
Raman spectroscopy of m-RGO LB film. (**a**) The bright field image of m-RGO LB film, (**b**) Raman mapping from the red box area of the bright field image, and (**c**) the averaged Raman spectrum of Raman mapping.

In Figure 7, an optical image of the m-RGO LB film deposited on slide glass, along with the corresponding Raman mapping and spectrum, is presented. The bright field image shown in Figure 7a clearly delineates the m-RGO LB film and glass area, with m-RGO sheets densely packed together. The Raman mapping image, obtained from the area within the red box in the bright field image, reflects the intensity of the D and G bands. The Raman mapping illustrates that m-RGO sheets are in close physical contact with each other. The Raman spectrum, obtained by averaging the D and G band intensities in the Raman mapping, reveals an I_D_/I_G_ ratio of around 1, indicating the presence of many sp3-hybridized carbon species in m-RGO resulting from the replacement of oxygen atoms in graphene oxide by sulfur atoms.

## 3. Experimental Section

### 3.1. Synthesis of Mercapto Reduced Graphene Oxide (m-RGO)

Graphene oxide (GO) was synthesized via a modified Hummers method from natural graphite flake (Sigma-Aldrich, St. Louis, MO, USA), as detailed in prior research conducted by our group [21,22]. Subsequently, mercapto reduced graphene oxide (m-RGO) was synthesized through a solvothermal reaction route, the comprehensive synthesis procedure of which was elucidated in our previous study [23]. In the typical synthesis process of m-RGO, a 30 mL suspension of GO (2 mg/mL) was subjected to a mixture with 10 mL of 1 M NaOH solution to induce agglomeration. The resulting agglomerated GO was then transferred to a centrifuge tube and centrifuged at 6000 rpm for 3 min, yielding GO sludge. The pH of the GO sludge was adjusted to approximately 9 through rinsing with deionized water accompanied by repeated centrifugation, followed by three washes with pyridine via vacuum filtration. The pyridine-washed GO sludge was subsequently re-dispersed in 30 mL of fresh pyridine and transferred to a Teflon-lined autoclave with a capacity of 45 mL. Subsequently, 650 mg of phosphorus decasulfide (P_4_S_10_, 99%, Sigma-Aldrich, St. Louis, MO, USA) was gradually added to the GO dispersion in pyridine. The assembled Parr bomb was then subjected to a heated dry oven at 180 °C for 6 h, followed by radiative cooling to room temperature. Following the reaction, the product underwent successive washes with deionized water and ethanol using vacuum filtration. The resultant product was then freeze-dried for characterization and subsequent applications.

### 3.2. Fabrication of m-RGO Langmuir–Blodgett (LB) Film

Equipment-free m-RGO LB films were fabricated using the “dropping and scooping method”, wherein m-RGO dispersion in ethanol was dropped onto water and subsequently transferred to a substrate. Initially, the m-RGO patches were invisible with the addition of a few drops of the m-RGO suspension. However, a very thin m-RGO Langmuir film began to appear on the water surface after approximately 0.3 mL of the m-RGO suspension was added. Upon continued addition of the m-RGO suspension to water, the m-RGO Langmuir film gradually spreads, eventually covering more than 95% of the water surface. To transfer the m-RGO Langmuir film to a substrate, we have employed methods such as gently scooping the m-RGO film or slowly draining the water.

### 3.3. Materials Characterization

X-ray photoelectron spectroscopic (XPS) measurements were carried out using a VG-220IXL spectrometer (Thermo Fisher Scientific, Waltham, MA, USA) with a monochromated Al Kα radiation (1486.6 eV, line width 0.8 eV). The pressure in the analysis chamber was about 10^−9^ torr while recording the spectra. The spectrometer had an energy resolution of 0.4 eV. All the binding energies were corrected with reference to C(1s) at 284.6 eV. Deconvolution of the spectrum was done using the CASA software (CASA XPS version 2.3.26) with an accuracy of 0.2 eV. The Shirley background was used for the deconvolution. Surface topography images were obtained using an atomic force microscope (AFM) (Pico-Plus AFM, Molecular imaging, Agilent technologies, Santa Clara, CA, USA). All AFM studies were performed in air using a tapping mode with SCANASYST-AIR tips (Bruker, Bilerica, MA, USA) The images were collected at a scan rate of 1.0 Hz in air. The Nano-Secondary Ion Mass Spectrometry (NanoSIMS, Cameca, Paris, France) measurements were done using a Cs^+^ primary ion beam. The beam current at the sample was lowered to ~0.48 pA by choosing a small diaphragm to get a much desired fine beam and high enough count rates for imaging of the sample. Negative secondary ions of ^12^C, ^16^O, ^32^S, and ^32^S^16^O were measured simultaneously using electron multipliers in the multicollection mode. Sufficient mass resolving power (MRP) to separate out mass interferences, e.g., ^32^S from ^31^P^1^H (MRP > 4000), was maintained by choosing an appropriate entrance slit. The typical measurement condition varied from 10 × 10 μm^2^ to 25 × 25 μm^2^ analysis areas and 10–15 layers. Each area was divided into 256^2^ pixels with dwell times of 40 ms/pixel. The ion images from multiple layers were corrected for beam drifts using the WinImage 8.10 software and the last 8–12 layers were added to form summed ion images.

## 4. Concluding Remarks

We have successfully demonstrated the facile, scalable, and reproducible fabrication of m-RGO Langmuir–Blodgett films on various substrates, including glass, mica, and Au film. The synthesized m-RGO contains a substantial number of thiol functional groups, which can alter the surface properties of the reduced graphene oxide. The sheet resistance of m-RGO films can be significantly reduced by increasing the film thickness, which can be controlled by adjusting the concentration of the m-RGO dispersion. Nano-SIMS elemental analysis revealed that thiol functional groups are well distributed on the m-RGO film. These thiol functional groups on the surface of the fabricated m-RGO film can be utilized for various applications, such as fabricating robust Au films on plastic substrates and developing biomolecule sensors using thiol as a cross-linker. Additionally, this simple and effective Langmuir–Blodgett film fabrication method may facilitate the commercial application of graphene films.

## Figures and Tables

**Table 1:** The influence of solvent on m-RGO Langmuir film formation at the water–air interface.

Solvent	Surface Tension (mN/m at 20 °C)	Density (g/mL)	Miscibility	Langmuir Film Formation
Ethanol	22.1	0.79	miscible	Very Good
Methanol	22.7	0.79	miscible	Very Good
Acetone	25.2	0.79	miscible	Very Good
Dimethylformamide	37.1	0.95	miscible	Very Good
N-Methyl-2-pyrrolidone	41	1.03	miscible	No Langmuir film forms
Dimethylsulfoxide	43.54	1.10	miscible	Not Good
Ethyl acetate	23.52	0.90	immiscible	Not Good
Butanol	24.21	0.81	immiscible	Not Good

Note: surface tension of water is 72.8 mN/m at 20 °C.

## Data Availability

The data that support the findings of this study are available from the corresponding authors upon reasonable request.
